# The Intersection of Race and Ethnicity, Nativity, and Sex on Cognitive Trajectories of Older Adults in the United States

**DOI:** 10.1093/geroni/igab046.925

**Published:** 2021-12-17

**Authors:** Marc Garcia, Wassim Tarraf

**Affiliations:** 1 Syracuse University, Syracuse, New York, United States; 2 Institute of Gerontology & Department of Healthcare Sciences, Wayne State University, Detroit, Michigan, United States

## Abstract

We used longitudinal data from the Health and Retirement Study (1998-2016) to estimate sex-specific age-graded changes in global cognition and memory among White, Black, and U.S.- and foreign-born Latino adults 51 years and older. Among males, racial/ethnic and nativity differences in cognitive function were mainly evident at younger ages, particularly for Blacks compared to Whites. We found no evidence to support male racial/ethnic or nativity differentials in trajectories of cognitive aging. For women, older Blacks and U.S.-Born Latinas, and to a lesser degree foreign-born Latinas, had lower cognitive function at younger ages. However, White women showed more pronounced cognitive aging in comparison to U.S.- and foreign-born Latinas. Results applied to both global and memory outcomes. Our findings support calls for nuanced considerations of racial/ethnic and nativity effects on cognitive aging and ADRDs. Continued monitoring of differential cognitive aging trends is warranted as the vascular and neurologic sequelae of COVID-19 manifests.

